# Regulation of Phytohormones on the Growth and Development of Plant Root Hair

**DOI:** 10.3389/fpls.2022.865302

**Published:** 2022-03-24

**Authors:** Mengxia Li, Yanchun Zhu, Susu Li, Wei Zhang, Changxi Yin, Yongjun Lin

**Affiliations:** ^1^National Key Laboratory of Crop Genetic Improvement, Huazhong Agricultural University, Wuhan, China; ^2^MOA Key Laboratory of Crop Ecophysiology and Farming System in the Middle Reaches of the Yangtze River, College of Plant Science and Technology, Huazhong Agricultural University, Wuhan, China; ^3^College of Life Science and Technology, Huazhong Agricultural University, Wuhan, China

**Keywords:** phytohormones, root hair cell fate determination, root hair initiation, root hair elongation, phytohormone interaction

## Abstract

The tubular-shaped unicellular extensions of plant epidermal cells known as root hairs are important components of plant roots and play crucial roles in absorbing nutrients and water and in responding to stress. The growth and development of root hair include, mainly, fate determination of root hair cells, root hair initiation, and root hair elongation. Phytohormones play important regulatory roles as signal molecules in the growth and development of root hair. In this review, we describe the regulatory roles of auxin, ethylene (ETH), jasmonate (JA), abscisic acid (ABA), gibberellin (GA), strigolactone (SL), cytokinin (CK), and brassinosteroid (BR) in the growth and development of plant root hairs. Auxin, ETH, and CK play positive regulation while BR plays negative regulation in the fate determination of root hair cells; Auxin, ETH, JA, CK, and ABA play positive regulation while BR plays negative regulation in the root hair initiation; Auxin, ETH, CK, and JA play positive regulation while BR, GA, and ABA play negative regulation in the root hair elongation. Phytohormones regulate root hair growth and development mainly by regulating transcription of root hair associated genes, including *WEREWOLF* (*WER*), *GLABRA2* (*GL2*), *CAPRICE* (*CPC*), and *HAIR DEFECTIVE 6* (*RHD6*). Auxin and ETH play vital roles in this regulation, with JA, ABA, SL, and BR interacting with auxin and ETH to regulate further the growth and development of root hairs.

## Introduction

The root system of a plant is an important structure in the organism. Its main functions are to fix the plant into the soil, obtain nutrients and water from the soil, and synthesize nitrogenous organic compounds such as amino acids. Root morphology and activity will directly affect the growth, development, and nutritional status of the whole plant (Yang et al., 2012). The root is composed of the root cap, the meristem zone, the elongation zone, and the mature zone (Bibikova and Gilroy, 2003). Root hairs are tubular-shaped unicellular extensions of epidermal cells, which serve to both expand the root surface area, facilitate the absorption and utilization of water and nutrients by the root system, and help plants respond to stress and interact with soil microorganisms (Peterson and Farquhar, 1996; Datta et al., 2011; Vissenberg et al., 2020).

The growth and development process of plant root hairs includes, mainly, root hair cell fate determination, root hair initiation, and root hair elongation (Bibikova and Gilroy, 2003). Root hair cells are derived from root epidermal cells, but not all epidermal cells can develop into root hairs. There are three types of root hair cell fate determination. The first type is random, in which all epidermal cells have the potential to differentiate into root hairs. Most dicotyledonous plants, ferns, and many monocotyledonous plants belong to this type (Clowes, 2000; Pemberton et al., 2001). The second type is asymmetrical cell differentiation, in which the asymmetric division of epidermal stem cells in the later meristems produces two different sizes of epidermal cells. Only the short epidermal cells can differentiate into root hairs. This type of differentiation mainly exists in some monocotyledons, primitive angiosperms, and nymphaeaceae plants (Kim and Dolan, 2011). The root hair cell fate determination method of *Arabidopsis* belongs to the third type, which is determined by the position effect. The epidermal cells in contact with two cortical cells (H-type cells) can develop to form root hairs. However, the epidermal cells connected to only one cortical cell (N-type cells) can often only develop into non-hair cells (Dolan et al., 1994).

The initiation of root hair refers to the process in which one side of the cell wall of the root epidermal cell with a special fate begins to expand, and the cell gradually widens and grows to form a tubular bulge. After the root hair cell completes the bulging process, it enters the elongation growth stage. When the root hair grows to a certain extent, the root hair will stop growing and enter the mature stage (Bibikova and Gilroy, 2003; Datta et al., 2011).

**Figure 1:**
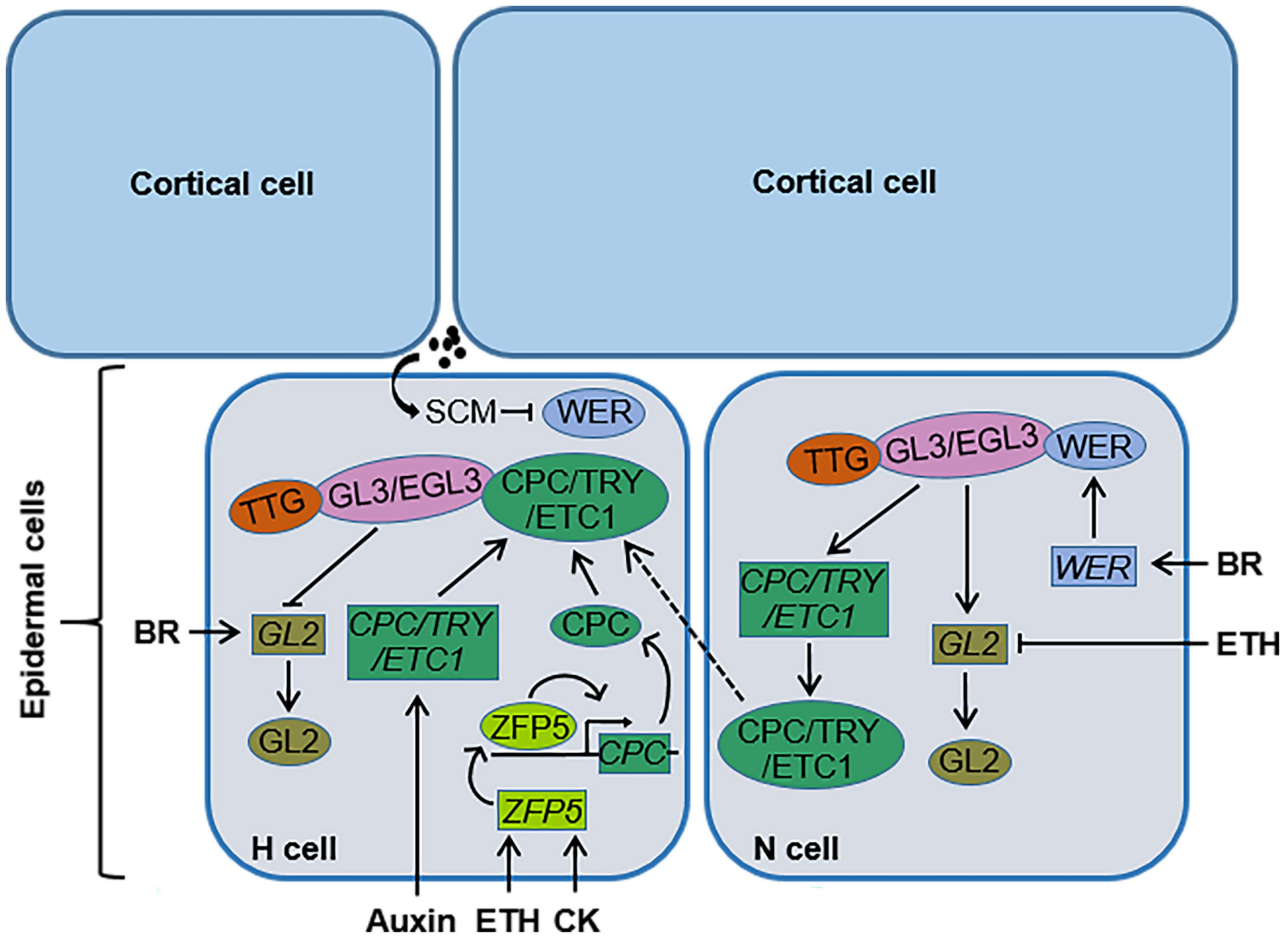
The regulation pattern of phytohormones on root hair cell fate determination in *Arabidopsis*. In N-type cells, WEREWOLF (WER), GLABRA3 (GL3), ENHANCER OF GLABRA3 (EGL3), and TRANSPARENT TESTA GLABRA (TTG) form a transcription complex WER-GL3/EGL3-TTG, which can induce the expression of *GLABRA2* (*GL2*) and *CAPRICE* (*CPC*) in N-type cells. GL2 determines the differentiation of N-type cells, and CPC can move laterally from N-type cells to neighboring H-type cells. BR is able to upregulate the expression of *WER*. In H-type cells, SCRAMBLED (SCM) can be specifically activated by indeterminate signals from the junction of two cortical cells, and this activated SCM can inhibit the accumulation of WER. CPC competes with WER to bind the complex GL3/EGL3-TTG. CPC and its functionally redundant homologs TRIPTYCHON (TRY) and ENHANCER OF TRY AND CPCs (ETC1) inhibit the expression of *GL2*, which regulates the differentiation of H-type cells. Cytokinin (CK) and ethylene (ETH) promote *CPC* transcription through transcription factor ZINC FINGER PROTEIN 5 (ZFP5), auxin upregulates the expression of *CPC*, *TRY*, and *ETC1*, and BR promotes the expression of *GL2* in H-type cells. ETH inhibits the transcription of *GL2* in N-type cells. →, positive regulation; ┤, negative regulation; →, transmembrane transport; ·, unknown signal. N cell, N-type cell; H cell, H-type cell; SCM, SCRAMBLED; BR, brassinosteroid; ETH, ethylene; CK, cytokinin.

**Figure 2:**
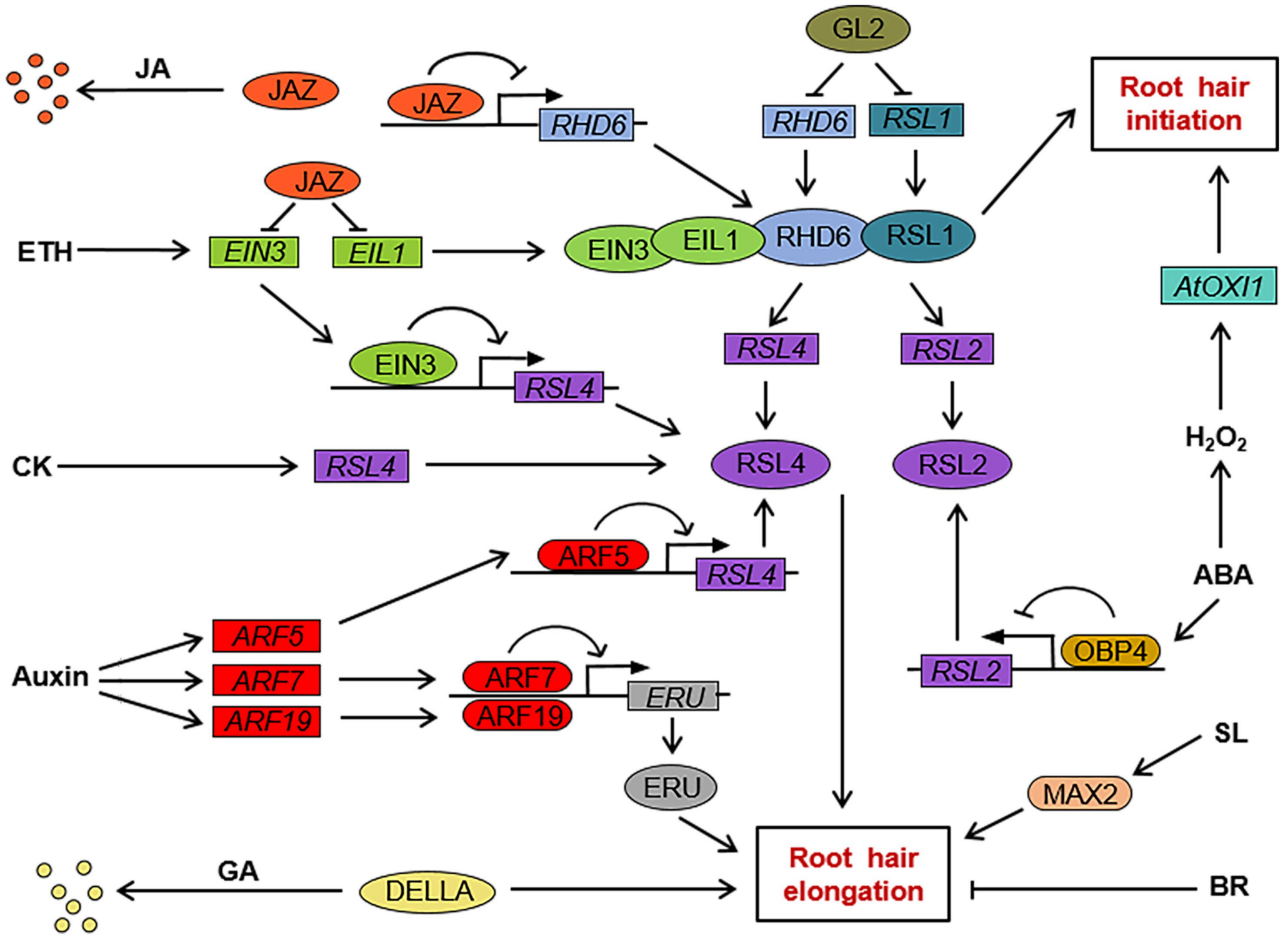
The regulation network of phytohormones on root hair initiation and elongation in *Arabidopsis*. *GL2* inhibits the expression of *ROOT HAIR DEFECTIVE 6* (*RHD6*) and RHD6 LIKE1 (*RSL1*), which are positive regulators of root hair initiation and elongation. RHD6 and RSL1 promote the expression of *RSL2* and *RSL4*, which in turn promote root hair elongation by upregulating the expression of elongation genes. JAZ protein, a repressor of JA signal transduction, inhibits *RHD6* transcription and interferes with the interaction between RHD6 and RSL1. JAZ protein also inhibits the transcription of *EIN3* and *EIL1* in the ethylene signaling pathway. EIN3/EIL1 can form a complex with RHD6/RSL1 to promote root hair initiation, and EIN3 binds to the promoter region of *RSL4* to promote its transcription. The auxin response factor ARF5 also binds to the *RSL4* promoter region to induce *RSL4* expression, and the auxin response factor ARF7 and ARF19 bind to the promoter region of *ERU* to induce *ERU* expression. GA negatively regulates the expression of root hair elongation genes through DELLA-mediated signal transduction. ABA induces the accumulation of OBP4 protein, and OBP4 binds to the *RSL2* promoter region to inhibit *RSL2* expression. ABA also induces the expression of *AtOXI1* and other root hair initiation-related genes by H_2_O_2_ production. CK can promote root hair elongation by promoting *RSL4* expression. SL induces root hair elongation-related gene expression through the MAX2-mediated signal transduction pathway. BR negatively regulates the expression of root hair elongation-related genes. →, positive regulation; ┤, negative regulation. JA, jasmonate; ETH, ethylene; CK, cytokinin; GA, gibberellin; BR, brassinosteroid; SL, strigolactone; and ABA, abscisic acid.

Root hair cell fate determination, root hair initiation, and root hair elongation are regulated by different genes. The molecular mechanism of root hair cell fate determination in the dicotyledonous model plant *Arabidopsis* has been relatively clear. The genes involved in root hair cell fate determination in root epidermal cells include *TRANSPARENT TESTA GLABRA* (*TTG*), *GLABRA3* (*GL3*), *ENHANCER OF GLABRA3* (*EGL3*), *WEREWOLF* (*WER*), *GLABRA2* (*GL2*) and *CAPRICE* (*CPC*), and its homologs *TRIPTYCHON* (*TRY*) and *ENHANCER OF TRY AND CPCs* (*ETC1*; Figure 1; Table 1; Galway et al., 1994; Rerie et al., 1994; Wada et al., 1997; Lee and Schiefelbein, 1999; Bernhardt et al., 2003; Shibata and Sugimoto, 2019). WER, GL3, EGL3, and TTG in N-type cells form a transcription complex, WER-GL3/EGL3-TTG, which accumulates in large amounts and can induce the expression of *GL2* and *CPC*. GL2 is a negative regulator of root hair growth and development and determines the differentiation of N-type cells (Rerie et al., 1994). CPC can move laterally from N-type cells to adjacent H-type cells (Wada et al., 1997). In H-type cells, CPC competes with WER to bind to the complex GL3/EGL3-TTG to inhibit the expression of GL2 and promote the differentiation of H-type cells (Shibata and Sugimoto, 2019). In addition, TRY and ETC1 play a functionally redundant role with CPC in H-type cells (Kirik et al., 2004; Simon et al., 2007). Downstream of *GL2*, bHLH transcription factors are involved in the regulation of root hair growth and development. *ROOT HAIR DEFECTIVE 6* (*RHD6*; Figure 2; Table 1) is a class I member of the Group VIII subfamily of bHLH, and plays a key role in regulating root hair initiation. In addition, RHD6 and its homolog RHD6 LIKE1 (RSL1) form a complex to regulate the expression of *RSL2* and *RSL4*, which are the secondary members of the Group VIII subfamily of bHLH. These secondary members play an active role in the process of root hair elongation (Masucci and Schiefelbein, 1994; Yi et al., 2010; Bruex et al., 2012; Shibata and Sugimoto, 2019). SCRAMBLED (SCM; Figure 1; Table 1) is a leucine-rich repeat receptor-like kinase (LRR-RLK), which can be specifically activated by as yet unidentified signals from the junction of two cortical cells. Activated SCM can promote the differentiation of H-type cells by reducing the accumulation of WER (Kwak and Schiefelbein, 2008). In contrast, only few advances in the molecular mechanism of root hair growth and development in crops have been made. It has been reported that rice genes, including *OsEXPB2* (Zou et al., 2015), *OsEXPA17* (Yu et al., 2011), *OsCSLD1* (Kim et al., 2007), *OsRHL1*, and *OsFH1* are involved in regulating rice root hair growth and development (Table 1; Ding et al., 2009; Huang et al., 2013). The *RTH* family genes in maize (Wen et al., 2005; Nestler et al., 2014) and the genes *HvEXPB1* and *HvEXPB7* in barely (Kwasniewski and Szarejko, 2006; He et al., 2015) are also involved in regulating the growth and development of root hair. However, there have been few studies on the mechanism of root hair growth and development in crop plants, including rice, maize, and barley, and the exact molecular regulatory network and regulatory mechanism of root hair growth and development are still unclear.

**Table 1:** The root hair growth and development-related genes in *Arabidopsis* and rice. –, negative regulation; +, positive regulation.

Genes	Accession Numbers	Root hair cell fate determination	Root hair initiation	Root hair elongation	References
*TTG*	At5g24520	−			Galway et al., 1994
*GL3*	At5g41315	−			Bernhardt et al., 2003
*EGL3*	At1g63650	−			Bernhardt et al., 2003
*WER*	At5g14750	−			Lee and Schiefelbein, 1999
*GL2*	At1g79840	−			Rerie et al., 1994
*CPC*	At2g46410	+			Wada et al., 1997
*TRY*	At5g53200	+			Kirik et al., 2004
*ETC1*	At1g01380	+			Kirik et al., 2004
*RHD6*	At1g66470		+		Masucci and Schiefelbein, 1996
*RSL1*	At5g37800		+		Bruex et al., 2012
*RSL2*	At4g33880			+	Shibata and Sugimoto, 2019
*RSL4*	At1g27740			+	Yi et al., 2010
*SCM*	At1g11130	+			Kwak and Schiefelbein, 2008
*OsEXPB2*	LOC_Os10g40710		+	+	Zou et al., 2015
*OsEXPA17*	LOC_Os06g01920			+	Yu et al., 2011
*OsCSLD1*	LOC_Os10g42750			+	Kim et al., 2007
*OsRHL1*	LOC_Os06g08500			+	Ding et al., 2009
*OsFH1*	LOC_Os01g67240			+	Huang et al., 2013
*ARF5*	At1g19850			+	Mangano et al., 2017
*ERU*	At5g61350			+	Schoenaers et al., 2018
*ARF7*	At5g20730			+	Schoenaers et al., 2018
*ARF19*	At1g19220			+	Schoenaers et al., 2018
*YUCCA*	At4g32540		+	+	Zhao et al., 2001
*AUX1*	At2g38120			+	Yu et al., 2015
*PIN2*	At5g57090		+	+	Cho et al., 2007
*EIN3*	At3g20770		+	+	Feng et al., 2017
*EIL1*	At2g27050		+	+	Feng et al., 2017
*ZFP5*	At1g10480		+	+	An et al., 2012
*MYB30*	At3g28910			+	Xiao et al., 2021
*OBP4*	At5g60850			−	Rymen et al., 2017
*AtOXI1*	At3g25250		+	+	Bai et al., 2007
*OsABIL2*	LOC_Os05g51510			−	Li et al., 2015a
*OsSAPK10*	LOC_Os03g41460			+	Yang et al., 2019
*OsAUX1*	LOC_Os05g37470			+	Jones et al., 2009
*GA20ox2*	At5g51810			−	Lv et al., 2018
*ACS2*	At1g01480			+	Yamagami et al., 2003
*TIR1*	At3g62980			+	Mayzlish-Gati et al., 2012
*BRI1*	At4g39400			+	Fridman et al., 2014
*AXR3/IAA17*	At1g04250		−	−	Kim et al., 2006

Increasing evidences reveals that phytohormones play important roles in regulating the growth and development of plant root hair. Phytohormones are trace organic compounds produced by the plant that can regulate plant growth and development at very low concentrations. Phytohormones can function at the synthetic site or be transported *via* the vascular system to distal tissues or organs (Jaillais and Chory, 2010; Wang and Irving, 2011). This review introduces the current research regarding auxin, ethylene (ETH), jasmonic acid (JA), abscisic acid (ABA), gibberellin (GA), strigolactone (SL), cytokinin (CK), and brassinosteroid (BR) in regulating the growth and development of plant root hairs. In addition, this review also discusses some unsolved questions in the field of phytohormone regulation and the molecular regulatory network of phytohormones. Progress into this field will provide theoretical basis and technical guidance for cultivating plants with well-developed root hairs.

## Auxin Regulation of the Growth and Development of Plant Root Hair

Auxin plays an essential role in the growth and development of plant root hair. Auxin is involved in regulating the determination of root hair cell fate and in promoting root hair initiation. Application of the exogenous auxin analogue naphthaleneacetic acid (NAA) can upregulate the expression of the *CPC*, *TRY*, and *ETC1* genes, which can positively determine the fate of root hair cells (Figure 1; Niu et al., 2011). CPC, TRY, and ETC1 compete with WER to bind the complex GL3/EGL3-TTG, inhibit the expression of *GL2*, and thus produce more root hairs. The auxin transport inhibitor N-1-naphthylphthalamic acid (NPA) can increase the expression of the negative regulation genes (*WER*, *GL3*, *GL2*, and *TTG*) of root hair cell fate determination and reduce the expression of the *TRY* gene, which then inhibits root hair initiation (Niu et al., 2011). In addition, *RHD6* positively regulates root hair initiation. Experimentally, the root hair density of the *rhd6* mutant was determined to be significantly lower than that of the wild type, and exogenous auxin treatment increased the root hair density of *rhd6*, indicating that auxin acts downstream of *RHD6* to regulate root hair initiation (Masucci and Schiefelbein, 1996).

Auxin is also involved in regulating the elongation of root hair. When compared to wild type *Arabidopsis*, the auxin-responsive mutant *axr1-12* had no difference in the number of root hairs but had significantly diminished lengths of root hairs, which indicates that auxin is necessary for the elongation of root hairs (Pitts et al., 1998). Exogenous auxin treatment can induce the expression of *RSL4* in roots and control the elongation of root hairs by regulating the expression of *RSL4* (Yi et al., 2010). Mangano et al. (2017) demonstrated that AUXIN RESPONSE FACTOR 5 (ARF5) was able to bind directly to the promoter of *RSL4* and induce the expression of *RSL4* (Figure 2; Table 1). In addition, *ERULUS* (*ERU*), a member of the *Catharanthus roseus* RECEPTOR-LIKE KINASE1-LIKE subfamily, controls cell wall formation during the elongation of root hair. AUXIN RESPONSE FACTOR 7 (ARF7) and AUXIN RESPONSE FACTOR 19 (ARF19) are both able to bind to the promoter region of *ERU* and regulate its expression to control root hair elongation (Figure 2; Table 1; Schoenaers et al., 2018).

The regulation of auxin on the growth and development of root hair is regulated by auxin metabolism and polar auxin transport, which controls the local auxin level and forms a concentration gradient. The formation of this auxin concentration gradient is the main mechanism involved in regulating many life activities (Marchant et al., 2002; Cho et al., 2007). There are two pathways for indole-3-acetic acid (IAA) synthesis in plants: the tryptophan pathway and the non-tryptophan pathway. During the process of IAA synthesis through the tryptophan pathway, YUCCA is the rate-limiting enzyme for converting indole-3-pyruvate (IPA) into IAA and is involved in regulating the initiation and the elongation of root hair. Previous studies demonstrated that overexpression of *yucca* in *Arabidopsis* increased the content of the endogenous auxin compared with that in the wild type, and the root hair density and length of transgenic plants were higher than that of the wild type (Zhao et al., 2001; Zhao, 2010). In addition, overexpression of *GmYUC2a*, a member of YUCCA gene family of soybean, significantly increased soybean root hair density (Wang et al., 2019).

Previous studies demonstrated that the auxin influx carrier AUX1 plays an important regulatory role in regulating root hair elongation in rice. Although *AUX1* is expressed in non-hair cells, but weakly or not at all in root hair cells (Jones et al., 2009), an *AUX1* gene mutation led to a significant inhibition of root hair elongation (Yu et al., 2015). There are differences in the expression patterns of *AUX1* in different plant roots. In addition, a mutation of the auxin efflux carrier gene *PIN2* inhibited the transport of auxin from the root tip to the root hair zone and inhibited the transport of auxin from non-hair cells to root hair cells. This resulted in the lack of auxin in root hair cells and inhibited the initiation and the elongation of root hair in *Arabidopsis* (Cho et al., 2007). These results indicated that non-hair cells may supply auxin to root hair cells and transport auxin to root hair cells in an AUX1 and PIN2 dependent manner (Cho et al., 2007; Jones et al., 2009).

## ETH Regulation of the Growth and Development of Plant Root Hair

Ethylene is involved in regulating root hair cell fate determination and in promoting root hair initiation. The number of root hairs in ETH-insensitive *Arabidopsis* mutant *etr1* is significantly lower than that in the wild type (Masucci and Schiefelbein, 1994). Exogenous application of the ETH biosynthesis inhibitor aminoethoxyvinylglycine (AVG) and the ETH action inhibitor Ag^+^ were able to significantly reduce the root hair density of the wild type, while the treatment of the ETH precursor 1-aminocyclopropane-1-carboxylic acid (ACC) increased the root hair density of the wild type (Masucci and Schiefelbein, 1994; Masucci and Schiefelbein, 1996; Dolan, 2001). Moreover, exogenous ACC treatment was also able to induce the formation of ectopic root hairs where non-treatment plants produced non-hair cells (Tanimoto et al., 1995). Recent study indicates that ETH induces the formation of ectopic root hairs by activating the transcription factor of ethylene signaling ETHYLENE INSENSITIVE 3 (EIN3)/EIN3-LIKE1 (EIL1) and reducing the transcription level of root hair growth inhibitor *GL2* (Qiu et al., 2021). EIN3 affects the formation of the WER-GL3-TTG1 complex by competing with GL3 for binding to TTG1, which in turn reduces the transcription of GL2 by decreasing the formation of the WER-GL3-TTG1 complex (Qiu et al., 2021). These results suggest that ETH is involved in regulating the fate determination of root hair cells, but the underling molecular regulation mechanism remains unclear.

Previous studies have shown that ETH may participate in the regulation of root hair cell fate determination and initiation through the C2H2 zinc finger protein ZINC FINGER PROTEIN 5 (ZFP5; Figure 1; Table 1). *ZFP5* is mainly expressed in the root and abundantly in root hair cells. The root hair density and root hair length of the *ZFP5* mutant *zfp5-4* were found to be lower than those of the wild type (An et al., 2012). Overexpression of *CPC* in the background of mutant *zfp5-4* restored the root hair phenotype of *zfp5-4* to the level of the wild type. The loss-of-function mutant *cpc* had nearly no root hair (An et al., 2012). However, overexpression of *ZFP5* in the background of *cpc* was not able to restore the defective root hair phenotype of *cpc*, indicating that CPC acts downstream of ZFP5 to regulate root hair cell fate determination and root hair initiation. Further studies showed that ZFP5 could directly bind to the promoter region of *CPC*, promote *CPC* transcription, and then participate in the fate determination of root hair cells and regulate the initiation and the elongation of root hair (Figure 1; An et al., 2012).

It has been reported that the root hair density of ETH insensitive mutants *etr1-1* and *etr1-3* was lower than that of the wild type and that the root hair density of ETH overproduction mutant *eto2* was higher than that of the wild type. In addition, the transcription of *ZFP5* in *etr1-1* and *etr1-3* was downregulated, and the transcription of *ZFP5* in *eto2* was upregulated. These results suggest that ETH participates in the regulation of root hair cell fate determination and root hair initiation by inducing the expression of *ZFP5* (An et al., 2012; Huang et al., 2020). Studies have also shown that when the fate determination mode of root hair cells is destroyed, the promoting effect of ETH on root hair initiation will be affected. When examining this, it was demonstrated that while the expression of *CPC* and *TRY* in root hair cells promoted the formation of root hairs, the number of root hairs of mutant *cpc* and double-mutant *cpc try* decreased significantly. Exogenous ETH treatment was able to significantly improve the root hair density of mutant *cpc*, but exogenous ETH treatment could not significantly improve the root hair density of double-mutant *cpc try*, indicating that ethylene can induce root hair initiation only when the CPC/TRY complex is functional or at least partially functional (Zhang et al., 2016).

Ethylene can promote the elongation of root hairs. The root hair length of the *Arabidopsis* ethylene-insensitive mutant *etr1* was lower than that of the wild type, while the root hair length of the ethylene overproduction mutant *eto1-1* was significantly longer than that of the wild type. Exogenous ACC treatment was able to increase the root hair length of the wild type and mutant *etr1* (Masucci and Schiefelbein, 1994; Pitts et al., 1998). ZFP5 is also involved in the process of ETH-promoted root hair elongation. The root hair length of the mutant *zfp5-4* was lower than that of the wild type. Exogenous ACC treatment was unable to restore the root hair length of *zfp5-4*, which indicates that ZFP5 is required for ETH to regulate *Arabidopsis* root hair elongation (An et al., 2012; Huang et al., 2020).

The key transcription factor EIN3 and its homologue EIL1 in the ethylene signaling pathway positively regulate the initiation and elongation of root hair (Figure 2; Table 1). The root hair density of the *Arabidopsis* double-mutant *ein3 eil1* was lower than that of the wild type, indicating that EIN3/EIL1 is involved in regulating root hair initiation. The root hair length of *ein3 eil1* was also shorter than that of the wild type, and it had no response to exogenous ACC treatment. These results indicate that EIN3/EIL1 is necessary for ETH to induce root hair elongation. EIN3 can directly bind to the promoter region of *RSL4* and activate transcription. EIN3 can also interact with RHD6 to activate the expression of *RSL4* and other related genes to promote root hair elongation (Feng et al., 2017).

In addition, MYB30, a member of MYB protein family, acts as a transcription factor in plants and negatively regulates the elongation of root hair (Table 1). MYB30 can directly bind to the promoter region of *RSL4* and inhibit its transcription, which then inhibits root hair elongation. ETH can promote EIN3 and MYB30 to form a complex by reducing the association between MYB30 and the *RSL4* promoter. This allows the transcription of *RSL4* to be activated, which promotes root hair elongation (Xiao et al., 2021).

Ethylene can interact with auxin to regulate the initiation and elongation of root hair (Figure 2; Muday et al., 2012). Compared with the wild type, the root hair length of the auxin-insensitive mutant *axr1* was significantly reduced, and exogenous ACC was able to restore the elongation of root hair. The ETH-insensitive mutant *ein2-1* was inhibited in root hair elongation, and exogenous NAA treatment was able to effectively alleviate this inhibition. The ETH overproducing mutant *eto1* had a long root hair phenotype, and the loss-of-function of AUX1 in the background of *eto1* reduced its root hair length (Pitts et al., 1998; Strader et al., 2010; Muday et al., 2012). In addition, transcriptome analysis revealed that auxin and ETH were able to upregulate the expression of 90% of the genes related to root hair growth and development (Bruex et al., 2012). These results show that auxin and ETH can regulate the growth and development of root hair in an interactive manner.

Ethylene and auxin do not have a simple upstream and downstream regulatory relationship, but it may be a mutual regulation model. IAA promotes ETH synthesis. Exogenous ETH also promotes IAA synthesis and upregulates the expression of IAA transport related genes to promote auxin transport to epidermal cells in the root elongation zone (Tsuchisaka and Theologis, 2004; Růžička et al., 2007). However, auxin and ETH signals do not regulate root hair growth and development in a completely interdependent manner. For example, exogenous application of IAA can increase the percentage of root hair cells among all epidermal cells in the double-mutant *aux1 etr1*, but ACC treatment has no such effect (Masucci and Schiefelbein, 1996). The above results show that auxin and ETH regulate root hair growth and development of plants by complex interactions and that the mechanisms of these interactions need to be further studied.

## JA Regulation of the Growth and Development of Plant Root Hair

Jasmonate plays a positive role in regulating the initiation and the elongation of root hair. It has been shown that exogenous treatment with appropriate concentration of JA and methyl jasmonate (MeJA) increased the root hair length and density in *Arabidopsis*. The increase in root hair density is induced by promoting more root hair cells to develop into root hair and by inducing the formation of branched hairs (Zhu et al., 2006). It has also been reported that exogenous application of MeJA significantly increased the root hair length. The COI1/JAZ-mediated JA signaling pathway is essential for the regulation of root hair elongation (Han et al., 2020). F-box protein COI1 is the receptor for JA, which positively regulates the JA response. JAZ proteins are repressors of JA signaling and can interact with a wide variety of transcription factors. The JAZ protein is degraded *via* the SCF^COI1^-26S proteasome pathway. The JA signal is then activated by releasing downstream transcription factors (Xie et al., 1998; Chini et al., 2007). The root hairs of the loss-of-function mutation of *COI1* and the *JAZ* overexpressed plants were much shorter than those of the wild type. The root hair length of the mutant containing five mutations in *jazQ* without JAZ function was longer than that of the wild type. The above results show that COI1/JAZ mediated JA signal transduction is necessary for root hair elongation. JAZ protein was able to interact with *RHD6* and *RSL1*, inhibit the transcription of *RHD6*, and interfere with the interaction between *RHD6* and *RSL1* (Figure 2). The phenotypic analysis showed that JA was involved in the regulation of root hair growth and development in a *RHD6* and *RSL1* dependent manner (Han et al., 2020). In addition, overexpression of cotton *GrTCP11* gene in *Arabidopsis* decreased the content of JA and the length of root hair compared with that of wild type. The results suggest that *GrTCP11* may regulate root hair elongation by negatively regulating the biosynthesis of JA and the responses of *Arabidopsis* to JA (Hao et al., 2021). However, it remains elusive whether *GrTCP11* regulates root hair elongation through a similar mechanism in cotton.

Jasmonate and ETH interact with each other to regulate the growth and development of root hairs. It has been reported that the promoting effect of JA on root hair initiation was weakened by ETH inhibitor (AVG or silver nitrate) and that the number of root hairs decreased significantly. Compared with the wild type, the promotion of MeJA and JA on root hair formation was also weakened in ETH insensitive mutants *etr1-1* and *etr1-3*. In addition, JA biosynthesis inhibitors ibuprofen and salicylhydroxamic acid (SHAM) treatment were able to inhibit the induction effect of ACC on root hair formation and reduce the root hair density of the ETH overproduction mutant *eto1-1*. These results suggest that JA promotes root hair initiation through its interaction with ETH (Zhu et al., 2006).

The ETH-activated transcription factor EIN3 and its homologue EIL1 were also identified as direct downstream targets of JAZ protein in the JA signaling pathway (Figure 2). The root hair density and lengths of the ETH double-mutant *ein3 eil1* were lower than those of the wild type. Exogenous application of JA was able to increase the root hair density and length of the wild type, but exogenous JA treatment was unable to increase the root hair density and length of *ein3 eil1*. Therefore, JA signal may positively regulate the fate determination and initiation of root hair cells through EIN3 and EIL1 (Zhu et al., 2011).

## ABA Regulation of the Growth and Development of Plant Root Hair

Previous research demonstrated that ABA positively regulates root hair cell fate determination and root hair initiation, but negative regulates root hair elongation. Exogenous ABA treatment increased root hair density not only by shortening the epidermal cells but also by inducing the formation of ectopic root hairs. However, the molecular mechanism by which ABA regulates root hair cell fate determination is still unclear (Lombardo and Lamattina, 2018). ABA plays a negative role in regulating root hair elongation. Exogenous ABA treatment leads to the shortening of root hair length of *Arabidopsis* seedlings. ABA induces the accumulation of OBF BINDING PROTEIN 4 (OBP4) protein, which directly inhibits the expression of *RSL2* and inhibits root hair elongation (Figure 2; Table 1; Schnall and Quatrano, 1992; Rymen et al., 2017).

Abscisic acid regulating root hair initiation partially depends on the H_2_O_2_. Exogenous application of H_2_O_2_ has been found to induce the production of more root hairs. However, there were significantly fewer root hairs in *Arabidopsis* treated with ABA plus H_2_O_2_ scavenger ascorbic acid (ASA) than in those treated with ABA alone. This result indicates that H_2_O_2_ is involved in the regulation of ABA on the initiation of root hairs (Bai et al., 2007). Previous results revealed that H_2_O_2_ can directly induce the expression of *AtOXI1*, which can positively regulate root hair initiation and elongation (Figure 2; Table 1). The root hair density and length of the loss-of-function mutant *atoxi1* were lower than those of the wild type. Exogenous ABA treatment was able to induce the expression of *AtOXI1* and promote root hair initiation. These results indicate that ABA-induced root hair initiation is mediated by H_2_O_2_ through the regulation of *AtOXI1* expression (Bai et al., 2007).

Previous studies demonstrated that ABA can positively regulate root hair initiation and root hair elongation in rice. Exogenous ABA treatment increased the area of the rice root hair zone, promoted the initiation of root hair, and then produced more root hair. ABA treatment also promoted rice root hair elongation (Chen et al., 2006; Wang et al., 2017). Further investigation indicated that ABA signal transduction may be involved in regulating rice root hair elongation. The *OsABIL2* gene is a member of the PP2C family and is a negative regulator in the ABA signal transduction pathway in rice. Overexpression of *OsABIL2* decreased the root hair length of transgenic rice compared with that of wild type rice (Table 1; Li et al., 2015a; Wang et al., 2017). SnRK2 is a positive regulator in the ABA signal transduction pathway, and *OsSAPK10* is a member of SnRK2 subclass III. Overexpression of *OsSAPK10* increased the root hair length of transgenic rice compared with that of wild type rice (Table 1; Wang et al., 2017; Yang et al., 2019).

Abscisic acid may participate in the regulation of rice root hair elongation by affecting auxin synthesis and polar auxin transport. It has been confirmed that ABA treatment can upregulate the expression of auxin synthesis genes including *YUCCAs* family members and auxin transport related genes such as *OsAUX1* and *OsPINs* in rice roots (Table 1). ABA may regulate auxin content by regulating the dynamic balance of auxin synthesis and transport, which then regulates rice root hair elongation (Wang et al., 2017).

In conclusion, ABA interacts with auxin in regulating the growth and development of root hair. ABA regulation on root hair growth and development can differ between species. For instance, ABA positively regulates the root hair cell fate determination and root hair initiation, negatively regulates root hair elongation in *Arabidopsis*, and positively regulates root hair initiation and root hair elongation in rice.

## GA Regulation of the Growth and Development of Plant Root Hair

Gibberellin positively regulates root hair initiation and negatively regulates root hair elongation. Previous results in *Brassica campestris* ssp. *chinensis* L. demonstrated that GA positively regulated root hair initiation. Exogenous GA_3_ treatment significantly increased the width of the root hair zone of *Brassica campestris* ssp. *chinensis* L. under saline alkali stress, and exogenous GA_3_ treatment increased the number of root hairs by promoting their initiation (Xu and Li, 2012).

Gibberellin negatively regulates root hair elongation (Figure 2). GA20-oxidase 2 (GA20ox2) is a rate-limiting enzyme of GA biosynthesis. The root hair length of transgenic *Arabidopsis* plants overexpressing the *GA20ox2* gene was shorter than that of the wild type (Table 1). Moreover, under salt stress, the expression of *GA20ox2* increased, GA_4_ accumulated, and root hair elongation was inhibited. The root hair length of transgenic plants with an overexpressed *GA20ox2* gene was shorter than that of the wild type. A loss-of-function mutant *ga20ox2-1* had longer root hairs than those of the wild type. These results suggest that GA20ox2 increases the content of endogenous active GA by promoting the synthesis of GA, which then negatively regulates the elongation of root hairs (Lv et al., 2018).

The inhibitory effect of GA on root hair elongation may be correlated to the DELLA protein. The DELLA protein is a negative regulator of GA signal transduction (Davière et al., 2008; Sun, 2011; Davière and Achard, 2013). The root hair length under low phosphorus conditions was much longer than that under normal culture conditions. Further investigation revealed that the content of bioactive GA decreased under low-phosphorus conditions, which leads to the accumulation of DELLA protein and inhibition of GA signal transduction (Jiang et al., 2007). These results indicate that GA negatively regulates root hair elongation through its biosynthesis and signal transduction.

## SL Regulation of the Growth and Development of Root Hair

Strigolactone has a positive regulatory effect on root hair elongation. MORE AXILLARY GROWTH2 (MAX2), an F-box protein, is an important component of the SL signal transduction pathway (Figure 2; Yao et al., 2017). Compared with the wild type, the root hair length of the SL signaling mutant *max2-1* was significantly reduced. Application of GR24, a synthetic strigolactone analog, to the wild type and SL synthesis deficient mutants resulted in an increase in root hair length, but application of GR24 to SL-insensitive *max2-1* did not restore its short root hair phenotype (Kapulnik et al., 2011a). These results indicated that SL promotes root hair elongation through MAX2-mediated signal transduction. In addition, overexpression of soybean *MORE AXILLARY GROWTH3* (*MAX3*) in *Arabidopsis* orthologs’ mutants can restore the phenotype of the root hair and enhance root hair elongation (Ul Haq et al., 2017). It is still unclear whether SL is involved in regulating the fate determination and/or the initiation of root hair cells.

Strigolactone interacts with ETH to regulate root hair growth and development. An SL signal mutant is sensitive to ETH. Application of ETH precursor ACC to the SL-insensitive *max2-1* was able to restore the short root hair of *max2-1*. In addition, the treatment of ETH biosynthesis inhibitor AVG was able to counteract the promoting effect of GR24 on root hair elongation of wild type plants. Therefore, ETH and SL may regulate root hair elongation in the same pathway, with ETH acting downstream of SL. ETH synthesis appears to be necessary for SL-mediated root hair elongation (Kapulnik et al., 2011b). Exogenous GR24 treatment can promote ETH biosynthesis by enhancing the transcription of *ACS2*, one of the rate-limiting enzymes of ETH biosynthesis, which then promotes root hair elongation (Table 1; Yamagami et al., 2003; Kapulnik et al., 2011b).

There are interactions between SL and auxin in regulating the growth and development of root hair. Application of IAA to the SL-insensitive mutant *max2-1* restored its short root hair phenotype. The root hair length of the auxin-insensitive deficient mutant *tir1-1* was shorter than that of the wild type. Exogenous GR24 treatment increased the root hair length of *tir1-1*. Simultaneous applications of IAA and GR24 to *Arabidopsis* resulted in a significant increase in the length of root hair, indicating that SL and auxin have an additive effect on promoting root hair elongation (Kapulnik et al., 2011b). The root hair density and length of tomato plant treated with excess GR24 were significantly lower than that of the control tomato plant. The inhibitory effect of excess GR24 on root hair density and length was counteracted by NPA, but not by exogenous IAA or NAA. Further investigation indicated that excessive SL in plants can promote the efflux of auxin, which decreases the auxin content of the plant, and thus inhibits the initiation and the elongation of root hair (Koltai et al., 2010). SL can also regulate the growth and development of root hair by regulating the expression of *PINs* (auxin polar transport genes) and *TIR1* (auxin receptor gene; Table 1; Koltai et al., 2010; Mayzlish-Gati et al., 2012; Zhang et al., 2020).

## CK Regulation of the Growth and Development of Plant Root Hair

Cytokinin plays a positive role in regulating the growth and development of root hair. Exogenous CK treatment can increase the density and the length of root hair (An et al., 2012; Zhang et al., 2016; Huang et al., 2020), but the underlying mechanism by which CK regulates root hair density and length is unclear. It has been reported that CK can regulate the fate determination, the initiation and the elongation of root hair cells through the C2H2 zinc finger protein ZFP5 (Figure 1; An et al., 2012). Exogenous synthetic CK 6-benzylaminopurine (6-BA) treatment was able to increase significantly the transcription level of *ZFP5* and increase the density of root hair. CK may regulate the fate determination of root hair cells and promote root hair initiation by regulating the expression of *ZFP5* (An et al., 2012). Exogenous CK treatment also caused an increase in the root hair length. Moreover, ZFP5 is involved in the process of CK promoting root hair elongation. The root hair length of the ZFP5 mutant *zfp5-4* was lower than that of the wild type, and exogenous 6-BA treatment was unable to restore the root hair length of *zfp5-4*, indicating that ZFP5 is necessary for CK to regulate *Arabidopsis* root hair elongation (An et al., 2012). In addition, exogenous CK treatment was also able to significantly increase the root hair length and density of oilseed rape (*Brassica napus* L.) and alfalfa (*Medicago sativa* L.; Silverman et al., 1998; Li et al., 2014).

Cytokinin may regulate root hair elongation through auxin and the ETH-independent pathway. Application of 6-BA treatment to the ETH insensitive mutant *etr1-1* and the auxin insensitive mutant *axr1* resulted in significant increases in root hair length for the two mutants. These results indicated that CK can promote root hair elongation in the absence of auxin or ETH signal. CK is not necessary for root hair elongation, as auxin and ETH can maintain root hair elongation in its absence (Zhang et al., 2016). Treatment with CK, auxin, and ETH alone can regulate the expression of genes related to root hair growth and development, and then regulate root hair elongation. For example, treatment of the mutant *rhd6* with CK, auxin, and ETH was able to induce the expression of *RSL4*, and then increase its root hair length (Masucci and Schiefelbein, 1994; Zhang et al., 2016).

## BR Regulation of the Growth and Development of Plant Root Hair

Brassinosteroid plays a negative role in regulating root hair cell fate determination and initiation (Figures 1, 2). Compared with the wild type, the number of root hairs of a BR signal deficient mutant increased. Following BR signal enhancement, the number of root hairs decreased. The expression levels of *WER* and *GL2* in the BR-insensitive mutant *bri1* were lower than in the wild type, while exogenous BR treatment was able to induce the expression of *WER* and *GL2* in *bri1*. BR can induce the expression of *WER*, which then promotes the formation of WER-GL3/EGL3-TTG complex, and consequently induces the expression of *GL2* and the differentiation of N-type cells. Enhancing the BR signal can promote the expression of *GL2* in root hair cells and inhibit the H-type cells from differentiating into root hair cells (Cheng et al., 2014; Wei and Li, 2016). However, the underlying molecular mechanism by which BR regulates the root hair cell fate determination is unclear and needs to be further studied.

Brassinosteroid regulation on root hair elongation differs among plant species. BR regulates root hair elongation in a concentration-dependent manner. It has been reported that BR negatively regulates root hair elongation and that the BR biosynthesis mutant *det2* had longer root hair when compared to the wild type. Treatment with synthetic brassinosterol analogue 24-epibrassinolide (24-epiBL) reduced the root hair length of *det2* to that of the wild type *Arabidopsis*. 24-epiBL treatment was able to significantly reduce the root hair length, while application of the BR synthesis inhibitor brassinazole (BRZ) promoted the elongation of root hair (Wang et al., 2010). Cell elongation has a great influence on the length of root hair, and the cell wall plasticity is an important determinant of cell elongation. Previous investigation revealed that, there, BR regulated root hair elongation in a cell-specific manner and that the overexpression of the BR receptor *BRASSINOSTEROID INSENSITIVE1* (*BRI1*) in H-type cells promoted the elongation of root hair cells. The expression of *BRI1* in non-hair cells inhibits cell elongation (Table 1). *BRI1* in non-hair cells activates the expression of ETH biosynthesis gene, resulting in the increase of ETH and the accumulation of cellulose in the cell wall of non-hair cells, thus inhibiting cell elongation (Fridman et al., 2014). In rice, there is a dose effect of the regulation of BR on root hair elongation. The treatment with lower concentrations of 24-epiBL promoted root hair elongation of the wild type rice. Increasing the 24-epiBL treatment concentration reduced its promotion of root hair elongation. When the concentration of 24-epiBL treatment reaches 1 μmol/L, it inhibits root hair elongation (Wang et al., 2010; Zhao et al., 2016).

The difference of BR regulation of root hair elongation between *Arabidopsis* and rice may be due to species specificity. For *Arabidopsis* root hair elongation, the endogenous BR content may be superoptimal. Consequently, exogenous BR synthesis inhibitor treatment may promote root hair elongation by reducing BR content, while any exogenous BR treatment will lead to an excessive concentration of BR and inhibit *Arabidopsis* root hair elongation. In contrast, for rice root hair elongation, the endogenous BR content may be lower than the optimal concentration. Thus, treatment with low concentration of BR can promote the elongation of rice root hair; however, treatment with high concentration of BR may lead to a superoptimal concentration of BR within the rice plant, leading to inhibition of root hair elongation.

Brassinosteroid interacts with auxin in the regulation of root hair initiation and elongation. Exogenous application of 24-epiBL upregulated the expression level of *AXR3/IAA17*, which plays a repressive role in auxin signal transduction. The initiation and the elongation of root hairs of transgenic plants overexpressing *AXR3/IAA17* were significantly inhibited (Table 1). In addition, the expression level of *AXR3/IAA17* in the BR-insensitive mutant *bri1* and the BR biosynthetic mutant *det2* decreased. These results suggest that BR may inhibit auxin signal transduction by promoting the expression of *AXR3/IAA17*, which then inhibits *Arabidopsis* root hair initiation and elongation (Kim et al., 2006). Additionally, exogenous application of BR can increase the length and the density of root hair by enhancing the transport of auxin *via* the regulation of AcPIN and AcAUX1/LAX in kiwifruit (Wu et al., 2021). These results indicate that there are interactions between BR and auxin in regulating the initiation and elongation of root hair.

## Conclusion and Perspective

Plant root hair can not only increase the absorption efficiency of water and nutrients in plants but can also enhance stress resistance and play an important role in regulating plant growth and development (Peterson and Farquhar, 1996; Bibikova and Gilroy, 2003; Datta et al., 2011; Vissenberg et al., 2020). Previous studies revealed that enhanced auxin transport under salt stress is able to promote the root hair growth and improve salt resistance of *Arabidopsis* (Fu et al., 2021). ABA accumulation can enhance drought resistance of barely by promoting root hair elongation (Zhang et al., 2021). In addition, elevated auxin concentration can enhance the resistance of tomato plant to early blight by promoting root hair production (Abdel-Motaal et al., 2020). Although it has been reported that *WER*, *TTG*, *GL3*, *GL2*, and *CPC* genes are involved in regulating the growth and development of root hair (Figures 1, 2), other genes that can directly regulate the growth and development of root hair have not yet been reported. Therefore, it is of great significance to identify genes that are involved in regulating root hair growth and development, and to investigate the regulatory functions of these unknown genes on root hair growth and development. Further work into this will make an important contribution to our full understanding of the molecular regulatory mechanism of root hair growth and development.

Although many important research advances have been made in the regulation of phytohormones on root hair initiation and elongation, there have been few studies on the regulation of phytohormones on root hair cell fate determination. It has not yet been reported whether JA, GA, and SL can regulate the root hair cell fate determination. The regulatory mechanism and effect of these phytohormones on the fate determination of root hair cells are still unclear.

There are complex interactions between phytohormones, which serve to jointly regulate the growth and development of root hair. Although it has been reported that JA, ABA, SL, and BR can regulate root hair growth and development by interacting with auxin or ETH, the mechanism of interaction remains to be fully described. For example, although previous results demonstrated that JA is able to mediate lateral root formation and taproot growth by regulating auxin synthesis, polar auxin transport, and signal transduction (Sun et al., 2009, 2011; Hentrich et al., 2013), it is unclear whether JA can also mediate root hair growth and development by regulating auxin synthesis, polar transport, and signal transduction. The molecular mechanism of the interaction between JA and auxin that regulates root hair growth and development needs to be further studied.

The molecular mechanism of auxin, SL, and GA in regulating root hair growth and development needs to be further studied. Previous studies revealed that auxin can promote rice internode elongation by promoting GA synthesis and inhibiting GA inactivation, which maintains a high content of active GA (Yin et al., 2007). GA can regulate root growth by affecting auxin transport and signal transduction, and auxin can also promote root growth by regulating the response of the root to GA (Fu and Harberd, 2003; Li et al., 2015b). However, during the regulatory process of root hair growth and development, the effect of the interaction between auxin and GA has not been reported, and the mechanism of interaction between auxin and GA on root hair growth and development is unclear. SL can regulate stem elongation by mediating GA metabolism and signal transduction (Zou et al., 2019), but the molecular mechanism of the interaction between SL and GA on root hair growth and development is not clear.

It has been reported that CK can promote ETH synthesis by increasing the stability of the rate-limiting enzyme ACS for ethylene biosynthesis (Hansen et al., 2009). The CK-induced ETH plays an important role in regulating the elongation of rice seminal root (Zou et al., 2018). However, it is unclear whether CK and ETH can interact to regulate root hair growth and development. In addition, it is unclear whether there are any interactions between JA, ABA, SL, BR, GA, and/or other phytohormones during the process of root hair growth and development and, if so, the molecular mechanism of these phytohormone interactions on root hair growth and development need to be further studied.

In addition, most of the current studies focus on the dicotyledonous model plant *Arabidopsis*, and there have been few studies on the regulation of phytohormones on the growth and development of root hairs of rice and other crops. In future research, identifying novel genes involved in regulating the growth and development of crop root hairs, investigating the biological functions of these genes, and exploring the regulatory effect and regulatory mechanism of phytohormones on root hair growth and development will provide theoretical basis and technical guidance for cultivating crops with developed root hairs. After exploring the new functions of known genes and identifying novel genes, it is possible to cultivate crops with well-developed root hairs by using the technologies including gene editing and genetic improvement. Cultivating and planting crops with well-developed root hairs can increase the efficiency of water and nutrient absorption by crops and enhance crop stress resistance, thereby increasing crop yields and ensuring world food security.

## Author Contributions

ML and CY conceptualized this manuscript and designed the graphs and table. ML wrote the original draft. YZ helped to write and revise the manuscript. YZ and CY revised and reviewed the format. SL and WZ assisted with the edited version. CY obtained funding. CY and YL contributed to final approval of the manuscript. All authors contributed to the article and approved the submitted version.

## Funding

This work was funded by the National Key Research and Development Program of China (No. 2016YFD0300102), the Science and Technology Major Projects of Guangxi Province of China (GuiKeChuang 010106), and the Postdoctoral Science Foundation of China (No. 2016T90705).

## Conflict of Interest

The authors declare that the research was conducted in the absence of any commercial or financial relationships that could be construed as a potential conflict of interest.

## Publisher’s Note

All claims expressed in this article are solely those of the authors and do not necessarily represent those of their affiliated organizations, or those of the publisher, the editors and the reviewers. Any product that may be evaluated in this article, or claim that may be made by its manufacturer, is not guaranteed or endorsed by the publisher.

## References

[ref1] Abdel-Motaal F. Kamel N. El-Zayat S. Abou-Ellail M. (2020). Early blight suppression and plant growth promotion potential of the endophyte *Aspergillus flavus* in tomato plant. Ann. Agric. Sci. 65, 117–123. doi: 10.1016/j.aoas.2020.07.001

[ref2] An L. Zhou Z. Sun L. Yan A. Xi W. Yu N.. (2012). A zinc finger protein gene *ZFP5* integrates phytohormone signaling to control root hair development in *Arabidopsis*. Plant J. 72, 474–490. doi: 10.1111/j.1365-313X.2012.05093.x, PMID: 22762888

[ref3] Bai L. Zhou Y. Zhang X. Song C. Cao M. (2007). Hydrogen peroxide modulates abscisic acid signaling in root growth and development in *Arabidopsis*. Chin. Sci. Bull. 52, 1142–1145. doi: 10.1007/s11434-007-0179-z

[ref4] Bernhardt C. Lee M. M. Gonzalez A. Zhang F. Lloyd A. Schiefelbein J. (2003). The bHLH genes *GLABRA3* (*GL3*) and *ENHANCER OF GLABRA3* (*EGL3*) specify epidermal cell fate in the *Arabidopsis* root. Development 130, 6431–6439. doi: 10.1242/dev.00880, PMID: 14627722

[ref5] Bibikova T. Gilroy S. (2003). Root hair development. J. Plant Growth Regul. 21, 383–415. doi: 10.1007/s00344-003-0007-x

[ref6] Bruex A. Kainkaryam R. M. Wieckowski Y. Kang Y. H. Bernhardt C. Xia Y.. (2012). A gene regulatory network for root epidermis cell differentiation in *Arabidopsis*. PLoS Genet. 8, e1002446–e1002420. doi: 10.1371/journal.pgen.1002446, PMID: 22253603 PMC3257299

[ref7] Chen C.-W. Yang Y.-W. Lur H.-S. Tsai Y.-G. Chang M.-C. (2006). A novel function of abscisic acid in the regulation of rice (*oryza sativa* L.) root growth and development. Plant Cell Physiol. 47, 1–13. doi: 10.1093/pcp/pci216, PMID: 16299003

[ref8] Cheng Y. Zhu W. Chen Y. Ito S. Asami T. Wang X. (2014). Brassinosteroids control root epidermal cell fate via direct regulation of a MYB-bHLH-WD40 complex by GSK3-like kinases. Elife 3:e02525. doi: 10.7554/eLife.02525, PMID: 24771765 PMC4005458

[ref9] Chini A. Fonseca S. Fernández G. Adie B. Chico J. M. Lorenzo O.. (2007). The JAZ family of repressors is the missing link in jasmonate signalling. Nature 448, 666–671. doi: 10.1038/nature06006, PMID: 17637675

[ref10] Cho M. Lee O. R. Ganguly A. Cho H. T. (2007). Auxin-signaling: short and long. J. Plant Biol. 50, 79–89. doi: 10.1007/BF03030615

[ref11] Clowes F. A. L. (2000). Pattern in root meristem development in angiosperms. New Phytol. 146, 83–94. doi: 10.2307/2588748

[ref12] Datta S. Kim C. M. Pernas M. Pires N. D. Proust H. Tam T.. (2011). Root hairs: development, growth and evolution at the plant-soil interface. Plant Soil 346, 1–14. doi: 10.1007/s11104-011-0845-4

[ref13] Davière J. M. Achard P. (2013). Gibberellin signaling in plants. Development 140, 1147–1151. doi: 10.1242/dev.087650, PMID: 23444347

[ref14] Davière J.-M. de Lucas M. Prat S. (2008). Transcriptional factor interaction: a central step in DELLA function. Curr. Opin. Genet. Dev. 18, 295–303. doi: 10.1016/j.gde.2008.05.004, PMID: 18590820

[ref15] Ding W. Yu Z. Tong Y. Huang W. Chen H. Wu P. (2009). A transcription factor with a bHLH domain regulates root hair development in rice. Cell Res. 19, 1309–1311. doi: 10.1038/cr.2009.109, PMID: 19752888

[ref16] Dolan L. (2001). The role of ethylene in root hair growth in Arabidopsis. J. Plant Nutr. Soil Sci. 164, 141–145. doi: 10.1002/1522-2624(200104)164:2<141::AID-JPLN141>3.3.CO;2-Q

[ref17] Dolan L. Duckett C. M. Grierson C. Linstead P. Schneider K. Lawson E.. (1994). Clonal relationships and cell patterning in the root epidermis of *Arabidopsis*. Development 8, 2241–2255. doi: 10.1101/gad.8.18.2241

[ref18] Feng Y. Xu P. Li B. Li P. Wen X. An F.. (2017). Ethylene promotes root hair growth through coordinated EIN3/EIL1 and RHD6/RSL1 activity in *Arabidopsis*. Proc. Natl. Acad. Sci. U.S.A. 114, 13834–13839. doi: 10.1073/pnas.1711723115, PMID: 29233944 PMC5748182

[ref19] Fridman Y. Elkouby L. Holland N. Vragovic K. Elbaum R. Savaldi-Goldstein S. (2014). Root growth is modulated by differential hormonal sensitivity in neighboring cells. Genes Dev. 28, 912–920. doi: 10.1101/gad.239335.114, PMID: 24736847 PMC4003282

[ref20] Fu X. Harberd N. P. (2003). Auxin promotes *Arabidopsis* root growth by modulating gibberellin response. Nature 421, 740–743. doi: 10.1038/nature01387, PMID: 12610625

[ref21] Fu J. Zhu C. Wang C. Liu L. Shen Q. Xu D.. (2021). Maize transcription factor ZmEREB20 enhanced salt tolerance in transgenic *Arabidopsis*. Plant Physiol. Biochem. 159, 257–267. doi: 10.1016/j.plaphy.2020.12.027, PMID: 33395583

[ref22] Galway M. E. Masucci J. D. Lloyd A. M. Walbot V. Davis R. W. Schiefelbein J. W. (1994). The *TTG* gene is required to specify epidermal cell fate and cell patterning in the *Arabidopsis* root. Dev. Biol. 166, 740–754. doi: 10.1006/dbio.1994.1352, PMID: 7813791

[ref23] Han X. Zhang M. Yang M. Hu Y. (2020). *Arabidopsis* JAZ proteins interact with and suppress RHD6 transcription factor to regulate jasmonate-stimulated root hair development. Plant Cell 32, 1049–1062. doi: 10.1105/tpc.19.00617, PMID: 31988260 PMC7145492

[ref24] Hansen M. Chae H. S. Kieber J. J. (2009). Regulation of ACS protein stability by cytokinin and brassinosteroid. Plant J. 57, 606–614. doi: 10.1111/j.1365-313X.2008.03711.x, PMID: 18980656 PMC2807401

[ref25] Hao J. Lou P. Han Y. Chen Z. Chen J. Ni J.. (2021). GrTCP11, a cotton TCP transcription factor, inhibits root hair elongation by down-regulating jasmonic acid pathway in *Arabidopsis thaliana*. Front. Plant Sci. 12:769675. doi: 10.3389/fpls.2021.769675, PMID: 34880892 PMC8646037

[ref26] He X. Zeng J. Cao F. Ahmed I. M. Zhang G. Vincze E.. (2015). *HvEXPB7*, a novel β-expansin gene revealed by the root hair transcriptome of Tibetan wild barley, improves root hair growth under drought stress. J. Exp. Bot. 66, 7405–7419. doi: 10.1093/jxb/erv436, PMID: 26417018 PMC4765802

[ref27] Hentrich M. Bottcher C. Duchting P. Cheng Y. F. Zhao Y. D. Berkowitz O.. (2013). The jasmonic acid signaling pathway is linked to auxin homeostasis through the modulation of YUCCA8 and *YUCCA9* gene expression. Plant J. 74, 626–637. doi: 10.1111/tpj.12152, PMID: 23425284 PMC3654092

[ref28] Huang L. Jiang Q. Wu J. An L. Zhou Z. Wong C.. (2020). Zinc finger protein 5 (*ZFP5*) associates with ethylene signaling to regulate the phosphate and potassium deficiency-induced root hair development in *Arabidopsis*. Plant Mol. Biol. 102, 143–158. doi: 10.1007/s11103-019-00937-4, PMID: 31782079

[ref29] Huang J. Kim C. M. Xuan Y.-H. Liu J. Kim T. H. Kim B.-K.. (2013). Formin homology 1 (OsFH1) regulates root-hair elongation in rice (*Oryza sativa*). Planta 237, 1227–1239. doi: 10.1007/s00425-013-1838-8, PMID: 23334469

[ref30] Jaillais Y. Chory J. (2010). Unraveling the paradoxes of plant hormone signaling integration. Nat. Struct. Mol. Biol. 17, 642–645. doi: 10.1038/nsmb0610-642, PMID: 20520656 PMC3166629

[ref31] Jiang C. Gao X. Liao L. Harberd N. P. Fu X. (2007). Phosphate starvation root architecture and anthocyanin accumulation responses are modulated by the gibberellin-DELLA signaling pathway in *Arabidopsis*. Plant Physiol. 145, 1460–1470. doi: 10.1104/pp.107.103788, PMID: 17932308 PMC2151698

[ref32] Jones A. R. Kramer E. M. Knox K. Swarup R. Bennett M. J. Lazarus C. M.. (2009). Auxin transport through non-hair cells sustains root-hair development. Nat. Cell Biol. 11, 78–84. doi: 10.1038/ncb1815, PMID: 19079245 PMC2635559

[ref33] Kapulnik Y. Delaux P.-M. Resnick N. Mayzlish-Gati E. Wininger S. Bhattacharya C.. (2011a). Strigolactones affect lateral root formation and root-hair elongation in *Arabidopsis*. Planta 233, 209–216. doi: 10.1007/s00425-010-1310-y, PMID: 21080198

[ref34] Kapulnik Y. Resnick N. Mayzlish-Gati E. Kaplan Y. Wininger S. Hershenhorn J.. (2011b). Strigolactones interact with ethylene and auxin in regulating root-hair elongation in *Arabidopsis*. J. Exp. Bot. 62, 2915–2924. doi: 10.1093/jxb/erq464, PMID: 21307387

[ref35] Kim C. M. Dolan L. (2011). Root hair development involves asymmetric cell division in *Brachypodium distachyon* and symmetric division in *Oryza sativa*. New Phytol. 192, 601–610. doi: 10.1111/j.1469-8137.2011.03839.x, PMID: 21848982

[ref36] Kim H. Park P.-J. Hwang H.-J. Lee S.-Y. Oh M.-H. Kim S.-G. (2006). Brassinosteroid signals control expression of the *AXR3/IAA17* gene in the cross-talk point with auxin in root development. Biosci. Biotechnol. Biochem. 70, 768–773. doi: 10.1271/bbb.70.768, PMID: 16636440

[ref37] Kim C. M. Park S. H. Il Je B. Park S. H. Park S. J. Piao H. L.. (2007). *OsCSLD1*, a cellulose synthase-Like D1 gene, is required for root hair morphogenesis in rice. Plant Physiol. 143, 1220–1230. doi: 10.1104/pp.106.091546, PMID: 17259288 PMC1820921

[ref38] Kirik V. Simon M. Huelskamp M. Schiefelbein J. (2004). The *ENHANCER OF TRY AND CPC1* gene acts redundantly with *TRIPTYCHON* and *CAPRICE* in trichome and root hair cell patterning in *Arabidopsis*. Dev. Biol. 268, 506–513. doi: 10.1016/j.ydbio.2003.12.037, PMID: 15063185

[ref39] Koltai H. Dor E. Hershenhorn J. Joel D. M. Weininger S. Lekalla S.. (2010). Strigolactones' effect on root growth and root-hair elongation may be mediated by auxin-efflux carriers. J. Plant Growth Regul. 29, 129–136. doi: 10.1007/s00344-009-9122-7

[ref40] Kwak S. H. Schiefelbein J. (2008). A feedback mechanism controlling SCRAMBLED receptor accumulation and cell-type pattern in *Arabidopsis*. Curr. Biol. 18, 1949–1954. doi: 10.1016/j.cub.2008.10.064, PMID: 19097902

[ref41] Kwasniewski M. Szarejko I. (2006). Molecular cloning and characterization of β-expansin gene related to root hair formation in barley. Plant Physiol. 141, 1149–1158. doi: 10.2307/20205836, PMID: 16679418 PMC1489888

[ref42] Lee M. M. Schiefelbein J. (1999). WEREWOLF, a MYB-related protein in *Arabidopsis*, is a position-dependent regulator of epidermal cell patterning. Cell 99, 473–483. doi: 10.1016/S0092-8674(00)81536-6, PMID: 10589676

[ref43] Li C. Shen H. Wang T. Wang X. (2015a). ABA regulates subcellular redistribution of OsABI-LIKE2, a negative regulator in ABA signaling, to control root architecture and drought resistance in *Oryza sativa*. Plant Cell Physiol. 56, 2396–2408. doi: 10.1093/pcp/pcv154, PMID: 26491145

[ref44] Li D. Shi T. Yuan P. Feng Y. Shi L. (2014). Differences in root microscopic structure of root mutants *lrn1*, *prl1* and wild type in oilseed rape (*Brassica napus* L.). Plant Sci. J. 32, 406–412. doi: 10.3724/SP.J.1142.2014.40406

[ref45] Li G. Zhu C. Gan L. Ng D. Xia K. (2015b). GA_3_ enhances root responsiveness to exogenous IAA by modulating auxin transport and signalling in *Arabidopsis*. Plant Cell Rep. 34, 483–494. doi: 10.1007/s00299-014-1728-y, PMID: 25540118

[ref46] Lombardo M. C. Lamattina L. (2018). Abscisic acid and nitric oxide modulate cytoskeleton organization, root hair growth and ectopic hair formation in *Arabidopsis*. Nitric Oxide - Biol. Chem. 80, 89–97. doi: 10.1016/j.niox.2018.09.002, PMID: 30236618

[ref47] Lv S. Yu D. Sun Q. Jiang J. (2018). Activation of gibberellin 20-oxidase 2 undermines auxin-dependent root and root hair growth in NaCl-stressed *Arabidopsis* seedlings. Plant Growth Regul. 84, 225–236. doi: 10.1007/s10725-017-0333-9

[ref48] Mangano S. Denita-Juarez S. P. Choi H.-S. Marzol E. Hwang Y. Ranocha P.. (2017). Molecular link between auxin and ROS-mediated polar growth. Proc. Natl. Acad. Sci. U.S.A. 114, 5289–5294. doi: 10.1073/pnas.1701536114, PMID: 28461488 PMC5441824

[ref49] Marchant A. Bhalerao R. Casimiro I. Eklöf J. Casero P. J. Bennett M.. (2002). AUX1 promotes lateral root formation by facilitating indole-3-acetic acid distribution between sink and source tissues in the *Arabidopsis* seedling. Plant Cell 14, 589–597. doi: 10.1105/tpc.010354.2, PMID: 11910006 PMC150581

[ref50] Masucci J. D. Schiefelbein J. W. (1994). The *rhd6* mutation of *Arabidopsis thaliana* alters root-hair initiation through an auxin- and ethylene-associated process. Plant Physiol. 106, 1335–1346. doi: 10.1104/pp.106.4.1335, PMID: 12232412 PMC159671

[ref51] Masucci J. D. Schiefelbein J. W. (1996). Hormones act downstream of *TTG* and *GL2* to promote root hair outgrowth during epidermis development in the *Arabidopsis* root. Plant Cell 8, 1505–1517. doi: 10.2307/3870246, PMID: 8837505 PMC161294

[ref52] Mayzlish-Gati E. De-Cuyper C. Goormachtig S. Beeckman T. Vuylsteke M. Brewer P. B.. (2012). Strigolactones are involved in root response to low phosphate conditions in *Arabidopsis*. Plant Physiol. 160, 1329–1341. doi: 10.1104/pp.112.202358, PMID: 22968830 PMC3490576

[ref53] Muday G. K. Rahman A. Binder B. M. (2012). Auxin and ethylene: collaborators or competitors? Trends Plant Sci. 17, 181–195. doi: 10.1016/j.tplants.2012.02.001, PMID: 22406007

[ref54] Nestler J. Liu S. Wen T.-J. Paschold A. Marcon C. Tang H. M.. (2014). *Roothairless5*, which functions in maize (*Zea mays* L.) root hair initiation and elongation encodes a monocot-specific NADPH oxidase. Plant J. 79, 729–740. doi: 10.1111/tpj.12578, PMID: 24902980

[ref55] Niu Y. Jin C. Jin G. Zhou Q. Lin X. Tang C.. (2011). Auxin modulates the enhanced development of root hairs in *Arabidopsis thaliana* (L.) Heynh. Under elevated CO_2_. Plant Cell Environ. 34, 1304–1317. doi: 10.1111/j.1365-3040.2011.02330.x, PMID: 21477123

[ref56] Pemberton L. M. S. Tsai S.-L. Lovell P. H. Harris P. J. (2001). Epidermal patterning in seedling roots of Eudicotyledons. Ann. Bot. 87, 649–654. doi: 10.1006/anbo.2001.1385

[ref57] Peterson R. L. Farquhar M. L. (1996). Root hairs: specialized tubular cells extending root surfaces. Bot. Rev. 62, 1–40. doi: 10.1007/bf02868919

[ref58] Pitts R. J. Cernac A. Estelle M. (1998). Auxin and ethylene promote root hair elongation in *Arabidopsis*. Plant J. 16, 553–560. doi: 10.1046/j.1365-313x.1998.00321.x, PMID: 10036773

[ref59] Qiu Y. Tao R. Feng Y. Xiao Z. Zhang D. Peng Y. (2021). EIN3 and RSL4 interfere with an MYB–bHLH–WD40 complex to mediate ethylene-induced ectopic root hair formation in *Arabidopsis*. Proc. Natl. Acad. Sci. U.S.A. 118:e2110004118. doi: 10.1073/pnas.2110004118, PMID: 34916289 PMC8713768

[ref60] Rerie W. G. Feldmann K. A. Marks M. D. (1994). The *GLABRA2* gene encodes a homeo domain protein required for normal trichome development in *Arabidopsis*. Genes Dev. 8, 1388–1399. doi: 10.1101/gad.8.12.1388, PMID: 7926739

[ref61] Růžička K. Ljung K. Vanneste S. Podhorská R. Beeckman T. Friml J.. (2007). Ethylene regulates root growth through effects on auxin biosynthesis and transport-dependent auxin distribution. Plant Cell 19, 2197–2212. doi: 10.1105/tpc.107.052126, PMID: 17630274 PMC1955700

[ref62] Rymen B. Kawamura A. Schäfer S. Breuer C. Iwase A. Shibata M.. (2017). ABA suppresses root hair growth via the OBP4 transcriptional regulator. Plant Physiol. 173, 1750–1762. doi: 10.1104/pp.16.01945, PMID: 28167701 PMC5338652

[ref63] Schnall J. A. Quatrano R. S. (1992). Abscisic acid elicits the water-stress response in root hairs of *Arabidopsis thaliana*. Plant Physiol. 100, 216–218. doi: 10.1104/pp.100.1.216, PMID: 16652949 PMC1075540

[ref64] Schoenaers S. Balcerowicz D. Breen G. Hill K. Zdanio M. Mouille G.. (2018). The auxin-regulated CrRLK1L kinase *ERULUS* controls cell wall vomposition during root hair tip growth. Curr. Biol. 28, 722–732.e6. doi: 10.1016/j.cub.2018.01.050, PMID: 29478854

[ref65] Shibata M. Sugimoto K. (2019). A gene regulatory network for root hair development. J. Plant Res. 132, 301–309. doi: 10.1007/s10265-019-01100-2, PMID: 30903397 PMC7082380

[ref66] Silverman F. P. Assiamah A. A. Bush D. S. (1998). Membrane transport and cytokinin action in root hairs of Medicago sativa. Planta 205, 23–31. doi: 10.2307/23385243

[ref67] Simon M. Lee M. M. Lin Y. Gish L. Schiefelbein J. (2007). Distinct and overlapping roles of single-repeat MYB genes in root epidermal patterning. Dev. Biol. 311, 566–578. doi: 10.1016/j.ydbio.2007.09.001, PMID: 17931617

[ref68] Strader L. C. Chen G. L. Bartel B. (2010). Ethylene directs auxin to control root cell expansion. Plant J. 64, 874–884. doi: 10.1111/j.1365-313X.2010.04373.x, PMID: 21105933 PMC3735369

[ref69] Sun T. (2011). The molecular mechanism and evolution of the GA–GID1–DELLA signaling module in plants. Curr. Biol. 21, R338–R345. doi: 10.1016/j.cub.2011.02.036, PMID: 21549956

[ref70] Sun J. Chen Q. Qi L. Jiang H. Li S. Xu Y.. (2011). Jasmonate modulates endocytosis and plasma membrane accumulation of the *Arabidopsis* PIN2 protein. New Phytol. 191, 360–375. doi: 10.1111/j.1469-8137.2011.03713.x, PMID: 21466556

[ref71] Sun J. Xu Y. Ye S. Jiang H. Chen Q. Liu F.. (2009). *Arabidopsis ASA1* is important for jasmonate-mediated regulation of auxin biosynthesis and transport during lateral root formation. Plant Cell 21, 1495–1511. doi: 10.1105/tpc.108.064303, PMID: 19435934 PMC2700526

[ref72] Tanimoto M. Roberts K. Dolan L. (1995). Ethylene is a positive regulator of root hair development in *Arabidopsis thaliana*. Plant J. 8, 943–948. doi: 10.1046/j.1365-313X.1995.8060943.x, PMID: 8580964

[ref73] Tsuchisaka A. Theologis A. (2004). Unique and overlapping expression patterns among the *Arabidopsis* 1-amino-cyclopropane-1-carboxylate synthase gene family members. Plant Physiol. 136, 2982–3000. doi: 10.1104/pp.104.049999, PMID: 15466221 PMC523360

[ref74] Ul Haq B. Ahmad M. Z. Rehman N. U. Wang J. Li P. Li D.. (2017). Functional characterization of soybean strigolactone biosynthesis and signaling genes in *Arabidopsis* MAX mutants and GmMAX3 in soybean nodulation. BMC Plant Biol. 17:259. doi: 10.1186/s12870-017-1182-4, PMID: 29268717 PMC5740752

[ref75] Vissenberg K. Claeijs N. Balcerowicz D. Schoenaers S. (2020). Hormonal regulation of root hair growth and responses to the environment in *Arabidopsis*. J. Exp. Bot. 71, 2412–2427. doi: 10.1093/jxb/eraa048, PMID: 31993645 PMC7178432

[ref76] Wada T. Tachibana T. Shimura Y. Okada K. (1997). Epidermal cell differentiation in *Arabidopsis* determined by a *Myb* homolog, *CPC*. Science 277, 1113–1116. doi: 10.1126/science.277.5329.1113 9262483

[ref77] Wang Y. H. Irving H. R. (2011). Developing a model of plant hormone interactions. Plant Signal. Behav. 6, 494–500. doi: 10.4161/psb.6.4.14558, PMID: 21406974 PMC3142376

[ref78] Wang T. Li C. Wu Z. Jia Y. Wang H. Sun S.. (2017). Abscisic acid regulates auxin homeostasis in rice root tips to promote root hair elongation. Front. Plant Sci. 8:1121. doi: 10.3389/fpls.2017.01121, PMID: 28702040 PMC5487450

[ref79] Wang F. Shi C. Dong J. (2010). The response of root hair on Brassinosteroids in *Arabidopsis* and rice. J. Agri. Univ. Hebei 33, 105–109. doi: 10.3969/j.issn.1000-1573.2010.06.021

[ref80] Wang Y. Yang W. Zuo Y. Zhu L. Hastwell A. H. Chen L.. (2019). *GmYUC2a* mediates auxin biosynthesis during root development and nodulation in soybean. J. Exp. Bot. 70, 3165–3176. doi: 10.1093/jxb/erz144, PMID: 30958883 PMC6598056

[ref81] Wei Z. Li J. (2016). Brassinosteroids regulate root growth, development, and symbiosis. Mol. Plant 9, 86–100. doi: 10.1016/j.molp.2015.12.003, PMID: 26700030

[ref82] Wen T.-J. Hochholdinger F. Sauer M. Bruce W. Schnable P. S. (2005). The *roothairless1* gene of maize encodes a homolog of *sec3*, which is involved in polar exocytosis. Plant Physiol. 138, 1637–1643. doi: 10.1104/pp.105.062174, PMID: 15980192 PMC1176433

[ref83] Wu Z. Gu S. Gu H. Cheng D. Li L. Guo X.. (2021). Physiological and transcriptomic analyses of brassinosteroid function in kiwifruit root. Environ. Exp. Bot. 194:104685. doi: 10.1016/j.envexpbot.2021.104685

[ref84] Xiao F. Gong Q. Zhao S. Lin H. Zhou H. (2021). MYB30 and ETHYLENE INSENSITIVE3 antagonistically modulate root hair growth in *Arabidopsis*. Plant J. 106, 480–492. doi: 10.1111/tpj.15180, PMID: 33529413

[ref85] Xie D.-X. Feys B. F. James S. Nieto-Rostro M. Turner J. G. (1998). *COI1*: An *Arabidopsis* gene required for jasmonate-regulated defense and fertility. Science 280, 1091–1094. doi: 10.1126/science.280.5366.1091, PMID: 9582125

[ref86] Xu F. Li T. (2012). Effects of exogenous GA_3_ on seed germination of *Brassica campestris* ssp. *chinensis* L. under mixed salt stress. Chin. J. Bioprocess Engineer. 10, 56–59. doi: 10.3969/j.issn.1672-3678.2012.06.012

[ref87] Yamagami T. Tsuchisaka A. Yamada K. Haddon W. F. Harden L. A. Theologis A. (2003). Biochemical diversity among the 1-amino-cyclopropane-1-carboxylate synthase isozymes encoded by the Arabidopsis gene family. J. Biol. Chem. 278, 49102–49112. doi: 10.1074/jbc.M308297200, PMID: 12968022

[ref88] Yang G. Yu Z. Gao L. Zheng C. (2019). SnRK2s at the crossroads of growth and stress responses. Trends Plant Sci. 24, 672–676. doi: 10.1016/j.tplants.2019.05.010, PMID: 31255544

[ref89] Yang J.-C. Zhang H. Zhang J.-H. (2012). Root morphology and physiology in relation to the yield formation of rice. J. Integr. Agric. 11, 920–926. doi: 10.1016/S2095-3119(12)60082-3

[ref90] Yao R. Li J. Xie D. (2017). Recent advances in molecular basis for strigolactone action. Sci. China Life Sci. 61, 277–284. doi: 10.1007/s11427-017-9195-x, PMID: 29116554

[ref91] Yi K. Menand B. Bell E. Dolan L. (2010). A basic helix-loop-helix transcription factor controls cell growth and size in root hairs. Nat. Genet. 42, 264–267. doi: 10.1038/ng.529, PMID: 20139979

[ref92] Yin C. Gan L. Ng D. Zhou X. Xia K. (2007). Decreased panicle-derived indole-3-acetic acid reduces gibberellin A1 level in the uppermost internode, causing panicle enclosure in male sterile rice Zhenshan 97A. J. Exp. Bot. 58, 2441–2449. doi: 10.1093/jxb/erm077, PMID: 17556768

[ref93] Yu Z. Kang B. He X. Lv S. Bai Y. Ding W.. (2011). Root hair-specific expansins modulate root hair elongation in rice. Plant J. 66, 725–734. doi: 10.1111/j.1365-313X.2011.04533.x, PMID: 21309868

[ref94] Yu C. Sun C. Shen C. Wang S. Liu F. Liu Y.. (2015). The auxin transporter, OsAUX1, is involved in primary root and root hair elongation and in Cd stress responses in rice (*Oryza sativa* L.). Plant J. 83, 818–830. doi: 10.1111/tpj.12929, PMID: 26140668

[ref95] Zhang S. Huang L. Yan A. Liu Y. Liu B. Yu C.. (2016). Multiple phytohormones promote root hair elongation by regulating a similar set of genes in the root epidermis in *Arabidopsis*. J. Exp. Bot. 67, 6363–6372. doi: 10.1093/jxb/erw400, PMID: 27799284 PMC5181580

[ref96] Zhang J. Mazur E. Balla J. Gallei M. Kalousek P. Medveďová Z.. (2020). Strigolactones inhibit auxin feedback on PIN-dependent auxin transport canalization. Nat. Commun. 11:3508. doi: 10.1038/s41467-020-17252-y, PMID: 32665554 PMC7360611

[ref97] Zhang Y. Xu F. Ding Y. Du H. Zhang Q. Dang X.. (2021). Abscisic acid mediates barley rhizosheath formation under mild soil drying by promoting root hair growth and auxin response. Plant Cell Environ. 44, 1935–1945. doi: 10.1111/pce.14036, PMID: 33629760

[ref98] Zhao Y. (2010). Auxin biosynthesis and its role in plant development. Annu. Rev. Plant Biol. 61, 49–64. doi: 10.1146/annurev-arplant-042809-112308, PMID: 20192736 PMC3070418

[ref99] Zhao Y. D. Christensen S. K. Fankhauser C. Cashman J. R. Cohen J. D. Weigel D.. (2001). A role for flavin monooxygenase-like enzymes in auxin biosynthesis. Science 291, 306–309. doi: 10.1126/science.291.5502.306, PMID: 11209081

[ref100] Zhao X. Wang Q. Yan Q. Zhao Y. Wang F. Dong J. (2016). Function of brassinolide in the regulation of root development in rice. Chin. J. Cell Biol. 38, 1191–1198. doi: 10.11844/cjcb.2016.10.0109

[ref101] Zhu Z. An F. Feng Y. Li P. Xue L. Mu A.. (2011). Derepression of ethylene-stabilized transcription factors (EIN3/EIL1) mediates jasmonate and ethylene signaling synergy in *Arabidopsis*. Proc. Natl. Acad. Sci. U.S.A. 108, 12539–12544. doi: 10.1073/pnas.1103959108, PMID: 21737749 PMC3145709

[ref102] Zhu C. Gan L. Shen Z. Xia K. (2006). Interactions between jasmonates and ethylene in the regulation of root hair development in *Arabidopsis*. J. Exp. Bot. 57, 1299–1308. doi: 10.1093/jxb/erj103, PMID: 16531464

[ref103] Zou X. Shao J. Wang Q. Chen P. Zhu Y. Yin C. (2018). Supraoptimal cytokinin content inhibits rice seminal root growth by reducing root meristem size and cell length via increased ethylene content. Int. J. Mol. Sci. 19:4051. doi: 10.3390/ijms19124051, PMID: 30558185 PMC6321243

[ref104] Zou X. Wang Q. Chen P. Yin C. Lin Y. (2019). Strigolactones regulate shoot elongation by mediating gibberellin metabolism and signaling in rice (*Oryza sativa* L.). J. Plant Physiol. 237, 72–79. doi: 10.1016/j.jplph.2019.04.003, PMID: 31026778

[ref105] Zou H. Wenwen Y. H. Zang G. Kang Z. Zhang Z. Huang J.. (2015). *OsEXPB2*, a β-expansin gene, is involved in rice root system architecture. Mol. Breed. 35, 1–14. doi: 10.1007/s11032-015-0203-y

